# Surveillance Studies Reveal Diverse and Potentially Pathogenic-Incriminated Vector Mosquito Species across Major Botswana Touristic Hotspots

**DOI:** 10.3390/insects12100913

**Published:** 2021-10-06

**Authors:** Mmabaledi Buxton, Casper Nyamukondiwa, Ryan J. Wasserman, Victor Othenin-Girard, Romain Pigeault, Philippe Christe, Olivier Glaizot

**Affiliations:** 1 Department of Biological Sciences and Biotechnology, Botswana International University of Science and Technology, P/Bag 016, Palapye 10071, Botswana; nyamukondiwac@biust.ac.bw (C.N.); ryanwas21@gmail.com (R.J.W.); 2 Department of Zoology and Entomology, Rhodes University, Makhanda 6140, South Africa; 3 Department of Ecology and Evolution, University of Lausanne, 1015 Lausanne, Switzerland; victor.othenin-girard@unil.ch (V.O.-G.); romain.pigeault@univ-poitiers.fr (R.P.); philippe.christe@unil.ch (P.C.); olivier.glaizot@unil.ch (O.G.); 4 EBI Ecologie & Biologie des Interactions (UMR 7267), Université de Poitiers, 86000 Poitiers, France; 5 Museum of Zoology, 1014 Lausanne, Switzerland

**Keywords:** Central Kalahari Game Reserve, Chobe enclave, emerging–re-emerging diseases, mosquito-borne infections, Okavango, vector mosquitoes

## Abstract

**Simple Summary:**

Mosquitoes vector pathogens that cause burdening diseases in humans, livestock and wildlife worldwide. Spatially and temporally, mosquito diversity varies considerably in response to bio-physical environments. As such, there is a need for mosquito diversity and distribution studies, as well as monitoring programmes, to inform on the risk of associated diseases. This survey assessed mosquito species in three major touristic areas of Botswana that are likely to harbour pathogens across prevailing hosts. The results revealed that all regions surveyed had important mosquito groups (*Anopheles*, *Aedes* and *Culex*) that are threats to public, wildlife and livestock health globally, including the arid Central Kalahari Game Reserve. The findings represent useful species inventories for future surveys and monitoring programmes.

**Abstract:**

Vector mosquitoes contribute significantly to the global burden of diseases in humans, livestock and wildlife. As such, the spatial distribution and abundance of mosquito species and their surveillance cannot be ignored. Here, we surveyed mosquito species across major tourism hotspots in semi-arid Botswana, including, for the first time, the Central Kalahari Game Reserve. Our results reported several mosquito species across seven genera, belonging to *Aedes*, *Anopheles*, *Culex*, *Mansonia*, *Mimomyia*, *Coquillettidia* and *Uranotaenia*. These results document a significant species inventory that may inform early warning vector-borne disease control systems and likely help manage the risk of emerging and re-emerging mosquito-borne infections.

## 1. Introduction

Arthropods are economically important organisms given their role in the global transmission of disease to humans, livestock and wildlife [1,2]. Amongst arthropods, mosquitoes are by far the most important vector species contributing to global human and animal health burdens [3,4]. Indeed, *Aedes*, *Anopheles*, *Culex* and *Mansonia* have been implicated as the main vector mosquito groups of medical and veterinary importance [5]. Vector mosquitoes transmit pathogens (e.g., viruses, protozoans and helminths) that cause diseases such as Zika, dengue, West Nile fever, yellow fever, Rift Valley fever, types of encephalitis (such as Japanese and St. Louis), chikungunya, filariasis and human and avian malaria [6,7,8,9]. Owing to the socio-economic burdens brought about by vector mosquitoes, adequate and updated knowledge on their species diversity, spatio-temporal distribution and abundance are thus key in monitoring and evaluating prevalence and the risk of their associated diseases.

The proliferation of mosquito vectors and associated pathogens poses a challenge in health and epidemiological systems, mainly in Africa [10]. Given the increase in invasive vector species with climate change [11], increased human populations, trade and globalisation [12,13], invasive vector mosquito species could exacerbate this problem. Indeed, mosquitoes contribute immensely to the global socio-economic burden of a global crisis in rising invasive insect species, and their economic costs are increasing significantly [14,15]. Mosquito species have the potential to successfully establish in diverse landscapes influenced by foraging (e.g., host availability, nectar dietary resources [16] or climatic niches [17]). Thus, in areas where there is a broad feeding preference (e.g., humans, livestock and wildlife), and favourable abiotic factors, mosquito species are likely to proliferate [18]. As such, novel environmental monuments such as game reserves and national parks with potentially diverse animal hosts, human influx, vegetation and perennial water supplies become a crucial ecological platform for vector–pathogen and host interaction fostering emerging and re-emerging mosquito-borne disease transmission dynamics [19]. Therefore, there is need for robust and continuous monitoring across major mosquito-vector-species risk pathways to update the inventory status [18].

In Africa, the most prominent mosquito-borne disease is malaria [20] (but also see Willcox et al. [21]; Mwanyika et al. [22]), accounting for ~400,000 deaths annually [23]. As such, most of the published works have mainly focused on *Anopheles* vector species and their pathogen incrimination surveillance [24] (but see Braack et al. [9]). Similarly, in Botswana much work is focused on human malaria vectors, neglecting other species [25]. In addition, mosquito research is also spatially skewed, mostly based in the malaria endemic zone (northern part) of the country and around permanent human habitation [26]. As such, there is a need for more holistic vector and pathogen surveillance that collectively covers all potential vector/parasite species and across different spatial scales.

Here, we assessed the diversity of mosquito vector species in major tourism hotspot regions of Botswana, including the Okavango Delta, the Chobe enclave and, for the first time, the Central Kalahari Game Reserve (CKGR). Currently, tourism is the second largest foreign exchange earner in Botswana, after diamonds [27]. Ecotourism comprises about 5% of the gross domestic product of the country’s economy [28], with the Okavango Delta, the Chobe enclave and the CKGR being the main centres of tourist activities [29]. Whilst previous works on mosquito diversity and vector competence have been conducted in parts of the Okavango and Chobe areas and other parts of the country [26,30,31] (see Table 1), there is still much exploration required given the potential anthropogenic and climate change likely to impact vector distribution and abundance [32]. This study thus provides an important species diversity database that may help strengthen vector surveillance, disease risk analysis and early warning systems in high-risk regions that are frequented by travelling humans.

## 2. Materials and Methods

### 2.1. Study Site and Mosquito Collection

**Figure 1:**
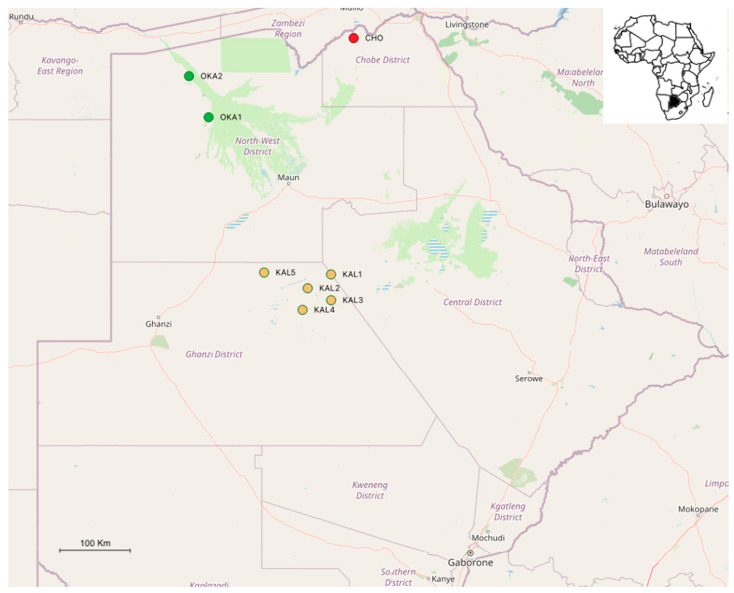
Map of Botswana with sampling stations: North-East Central Kalahari Game Reserve (CKGR)—KAL1 to KAL5; Okavango Delta—OKA1 and OKA2; and the Chobe enclave—CHO. See Table 2 for details of stations. Thumbnail picture shows the location of Botswana in Africa.

Sampling was done in March 2019 under Ministry of Environment, Natural Resources Conservation & Tourism of the Republic of Botswana research permit number ENT 8/36/4 XXXX II (10/82). Adult mosquitoes were sampled around 5 artificial water holes in the north-east of the Central Kalahari Game Reserve (CKGR, Ghanzi District, 1 night per station, Figure 1) as well as in the Chobe enclave, at the VanThuyne-Ridge (VTR) Research Centre (Chobe District, 5 nights, Figure 1).

In February 2020, 2 stations west of Okavango (North-West District) were prospected during 3 and 2 nights, respectively. Sampling was done again at the VTR Research Centre for 5 nights (Figure 1; Table 2). All samples were collected during the summer season of the southern hemisphere, when vector mosquitoes are most abundant [38].

The CKGR is a hot desert region with a distinct dry (April to October) and wet (November to March) season. Semi-permanent but scarce artificial water bodies are found across the region. During the wet season, the landscape becomes green and dry with no rains [39]. The Okavango Delta is largely characterised by permanent streams and swampy basins to seasonal floodplains. Although the wet and dry seasons follow the same pattern as the CKGR, the Okavango Delta is rich in fauna and flora; however, both regions remain unique in attracting tourists from diverse local and international destinations [40]. Whilst the Chobe enclave consists of permanent river systems in the northern part of the district, which sustain wildlife, the southern region is relatively dry [41]. Sampling was carried out on the dry area (southern part) away from the Chobe River, i.e., around the VTR Centre (9 km south of Parakarungu village). Whilst the Chobe enclave and the Okavango Delta are largely characterised by a livestock and wildlife interface, the CKGR site is predominantly characterised by wildlife [42].

Six BG-Sentinel carbon-dioxide-baited traps (Biogents AG, Regensberg, Germany) were set in the afternoon (1600 h) and the mosquitoes were collected in the morning (0600 h). The specimens were preserved individually in 2 mL Eppendorf tubes with 80% ethanol. However, because of overwhelming adult mosquito catches in the Okavango (up to 1500/night), the mosquitoes were first separated into their genera in the field using gross morphology [43] and then stored by identified taxon in 25 or 50 mL vials.

### 2.2. Sample Identification and Analyses

All specimens were identified morphologically following the protocols of Jupp [43] under a stereo binocular microscope (Leica M205, Leica microsystems, Wetzlar, Germany). Further, 1 to 40 samples per taxon, depending on the number of individuals collected, were subjected to molecular identification following Buxton et al. [36]. Here, the cytochrome oxidase subunit I (COI) gene was amplified using LCO1490 (5′-GGTCAACAAATCATAAAGATATTGG-3′) and HCO2198 (5′-TAAACTTCAGGGTGACCAAAAAATCA-3′) primers [44]. Sequences were examined with the BLAST algorithm in GenBank (accessed in June 2021). Molecular identification was used whenever blast identity was above 98%. When sequences were not available in GenBank (or blast identity was ≤ 98%), we used morphological identities. Unidentified specimens (morphological and molecular) were given to the genera level by groups of morphologically identical specimens (e.g., sp. 1, sp. 2).

Mosquito species richness (i.e., total species represented) per site was determined and abundances were analysed as proportions using percentages across sampled areas to compare dominance. The Shannon–Wiener diversity index was determined (equation 1), which takes into account the species abundance and evenness (i.e., the proportion of individuals amongst the different species sampled) following protocols of Khoobdel et al. [45]. Thus, the Shannon diversity index (H′) was calculated as the summation of the product of species abundance probability (*Pi*) and its natural logarithm (*lnPi*).

*H*′ = −∑*Pi* · *ln*(*pi*) (1)

## 3. Results

**Figure 2:**
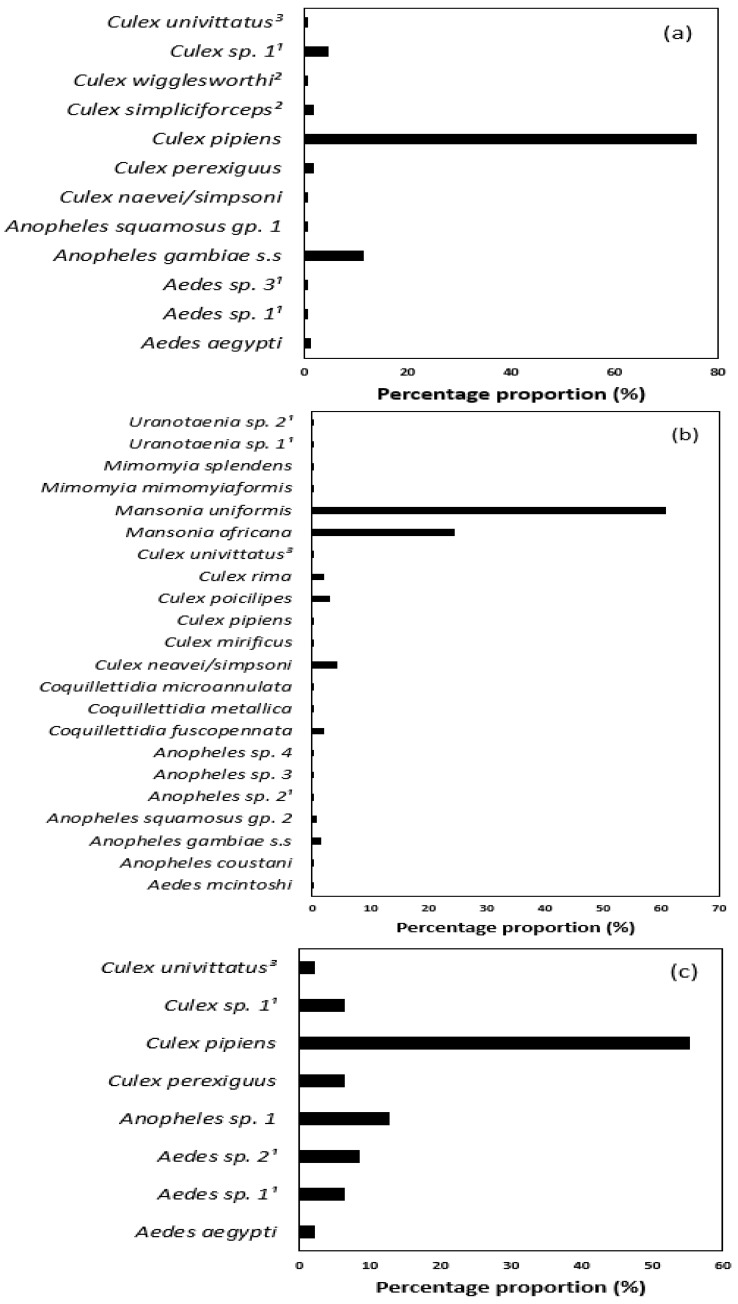
Summary of the diversity proportions (percentage) of mosquito species sampled from the (**a**) Chobe enclave, (**b**) Okavango and (**c**) Central Kalahari Game Reserve (CKGR) between March 2019 and February 2020.

A total of 5486 mosquitoes from 32 taxa of seven genera (*Aedes*, *Anopheles*, *Culex*, *Mansonia*, *Mimomyia*, *Coquillettidia* and *Uranotaenia*) were collected (Table 3). In the Chobe enclave (CHO), 173 mosquitoes of 9 species in 3 genera were recorded (*Aedes*: *Ae. aegypti*; *Anopheles*: *An. gambiae* s.s, *An. squamosus* gp. 1; and *Culex*: *Cx. naevei/simpsoni*, *Cx. perexiguus*, *Cx. pipiens*, *Cx. simpliciforceps*, *Cx. wigglesworthi* and *Cx. univittatus*). Two unidentified *Aedes* specimens (*Ae.* sp. 1^1^ and *Ae*. sp. 3^1^) and eight unidentified *Culex* (*Cx.* sp. 1^1^) were collected in Chobe as well (Table 3). *Culex pipiens* was the most dominant species (75.72%), followed by *An. gambiae* s.s (11.56%). Other mosquito taxa reported were few, ranging from one (0.58%) to eight (4.62%) specimens per taxa (Figure 2a).

The Okavango Delta (OKA) was by far the richest region with 5266 mosquitoes collected (96% of all catches), representing 17 mosquito species and a further 5 unidentified taxa (Table 3, Figure 2b). The area was largely dominated by two *Mansonia* species (*Ma. uniformis* and *Ma. africana*), which account for 60.9% and 24.5% of the mosquitoes caught in the region, respectively. Other frequent species were *Cx. naevei/simpsoni* (4.35%) and *Cx. poicilipes* (3.11%). Seven other species of *Culex* were caught in the station with lower abundances, ranging from 1 to 111 individuals (Table 3). The proportion of *Cx. pipiens* (less than 1%) was relatively low compared to their frequency in other regions. Other species were *Ae. mcintoshi*, seven species of *Anopheles* (*An. gambiae* being the most prevalent with 1.41% of the catches), three species of *Coquillettidia* (*Cq. fuscopennata*, *Cq. metallica*, *Cq. microannulata*), two species each of *Mimomyia* (*Mi. mimomyiaformis*, *Mi. splendens*) and *Uranotaenia* (*Uranotaenia* sp. 1, *Uranotaenia* sp. 2) (Table 3).

The Central Kalahari Game Reserve (CKGR) reported one species of *Aedes* (*Ae. aegypti*, 2.13%) and three species of *Culex* (*Cx. univittatus*, *Cx. perexiguus* and *Cx. pipiens*) representing 2.13%, 6.38% and 55.32% of the specimens collected, respectively (Table 3, Figure 2c). It was the least diverse region sampled, and the majority and most abundant species (*Cx. pipiens*) was collected at the gate (CKGR_1), where the rangers’ camp was located. Other individuals were of three unidentified species of *Aedes* and one species of *Culex*, all being relatively rare (three to five specimens).

Okavango reported the highest species richness (22), the Kalahari had the lowest (8) and Chobe was intermediate (12). The species diversity index was highest in the Kalahari (1.491), whereas Chobe had the lowest diversity (0.973) with Okavango being intermediate (1.207). Overall, all the sampled areas had globally important mosquito genera of medical and veterinary importance (*Aedes*, *Anopheles* and *Culex*) with *Cx. pipiens* species reported in all study sites (Table 4).

## 4. Discussions

The current study documents 32 mosquito taxa (22 identified at the species level and 10 at the genera level) belonging to 7 genera in Botswana’s tourism hotspots. To the best of our knowledge, this is the first study to date documenting mosquito species from the Kalahari Desert and one of the few in other major touristic hotspots in Botswana (but see Pachka et al. [30]; Cornel et al. [31]). Congruent to Cornel et al. [31], we found mosquito species of important medical and veterinary concern (see Table 4). In particular, the results showed that *Cx. pipiens* dominated other species in the Chobe enclave and CKGR. Most of the *Cx. pipiens* in CKGR were collected in a station next to the park entrance (CKGR_1, Figure 1) where people from the park are living, in accordance with its anthropogenic habits such as the colonisation of sewage systems. *Mansonia uniformis* and, to a lesser extent, *Ma. africana* by far dominated the Okavango area. *Mansonia uniformis* is known to feed mostly on mammals (e.g., cattle, humans) but readily bites other vertebrate hosts, including avian species [64,65]. The alarming high numbers of the duo species should serve as a potential early warning sign of the risk of emerging–re-emerging diseases and pathogen circulation across an interface of humans, livestock and wildlife in the region. This study is also the first to report on the invasive species *Ae. aegypti* in the Chobe enclave and in the CKGR. It has been observed in Botswana for the first time in the south-east of the country, close to the South African border [36]. Apart from transmitting several pathogens of medical importance (e.g., Zika, dengue, yellow fever and chikungunya viruses), *Ae. aegypti* has also been reported to transmit lumpy skin virus in cattle [47] and potentially to wildlife. The unidentified species of *Aedes* (and other unidentified taxa) have implications for the risk of associated diseases in touristic areas; moreover, their pathogenicity status has not yet been assessed. Furthermore, we also report mosquito species belonging to *Coquillettidia*, *Mansonia*, *Mimomyia* and *Uranotaenia* only in the swampy Okavango, suggesting spatial environmental context effects in determining mosquito species diversity, given the highest richness reported in the area [66].

Differences between the three study sites are likely driven by differences in environmental conditions. The CKGR is more arid, with no permanent water sources supporting mosquito breeding, than to the Chobe enclave and Okavango. Thus, mosquito breeding in the CKGR during dry seasons may be restricted to artificial water holes designed to water wildlife. This may partly account for the fewer mosquito species recorded in the CKGR. Conversely, Okavango consists of semi-permanent basins that are likely to sustain mosquito-breeding success, leading to elevated species richness in the area. For instance, *Coquillettidia, Mimomyia* and *Mansonia* species were reported only in the Okavango likely supported by the swampy wetland systems with large clear water bodies, whereby their larvae attach to the submerged aquatic plants for sustained feeding and oxygen intake [67]. The Chobe enclave has permanent water bodies likely to support mosquito species, congruent with Buxton et al. [33]. However, sampling was performed in a relatively arid part of the Chobe enclave, where breeding opportunities are mostly related to the presence of human infrastructure, as indicated by the majority of anthropogenic species found in this region (*Ae. aegypti, An. gambiae* s.s and *Cx. pipiens*). Large water bodies, through their semi-permanent swamps, and water that collects in animal hoof prints are a major source of facilitating mosquito-breeding refuge [33,68]. Although habitat characterisation was not included in this study, resource portioning and species distribution remains critical for mosquito vector success. This has an implication on the risk of pathogens they may potentially transmit and the distribution of ‘previously unidentified’ mosquitoes to species level across touristic regions. It is well known that increasing human populations, international travel and globalisation have implications on importing and/or exporting vectors, pathogens and parasites from native ranges or areas of most prevalence [69]. Despite this, however, mosquito diversity from tourism hotspots in Botswana remains relatively unexplored [30,31,70].

The presence of numerous vector species in tourist areas in Botswana, including the invasive *Ae. aegypti*, a major contributor of dengue infections in the tropics and subtropics globally [71], highlights the need for rigorous mosquito vector surveillance in these areas. Therefore, the use of different trapping techniques in time and space may be essential in exploring a full spectrum of mosquito species diversity across varying seasonal and environmental niches [72]. More robust and spatially even sampling protocols with standardised effort may also be needed to get more accurate diversity indices across the studied sites. Furthermore, pathogen screening in vectors and prevailing vertebrate hosts (humans, livestock and wildlife) is pivotal in assessing transmission dynamics and the potential risk of emerging and re-emerging diseases in the country. This study also reported *Cx. pipiens* mosquitoes, which exist as a species complex. Thus, future studies may explore genetic variation among sibling species and consider surveillance of the diversity of other arthropod vectors capable of incriminating humans, livestock and wildlife with their associated pathogens. This study nevertheless provides significant baseline vector diversity data that may play a significant role in identifying infection risk pathways in pest and disease risk analysis and under emerging and re-emerging vector-borne infections.

## Figures and Tables

**Table 1:** Identified mosquito species in Botswana belonging to genera *Anopheles*, *Aedes*, *Culex* and *Mansonia*.

*Anopheles*	Aedes	Culex	Mansonia
*An. arabiensis*	*Ae. mcintoshi*	*Cx. pipiens*	*Ma. uniformis*
*An. parensis*	*Ae. aegypti*	*Cx. poicillipes*	
*An. longipalpis* type C		*Cx. neavei*	
*An. leesoni*		*Cx. antennatus*	
*An. quadriannulatus*			
*An. funestus s.s*			
*An. rivulorum*			
*An. pharoensis*			
*An. nili*			
*An. rufipes*			
*An. distinctus*			
*An. squamosus*			
*An. ziemanni*			
*An. demeilloni*			
*An. marshalli*			
*An. vaneedeni*			
*An. rhodesiensis*			
*An. seretsei*			
Refs [26,31,33,34,35]	[31,36]	[30,31,37]	[31]

**Table 2:** Locations and mosquito sampling dates across areas of touristic destinations in Botswana between March 2019 and February 2020.

Location	Site	Sampling Dates	Station Code	GPS Coordinates
Chobe enclave	VanThuyne-Ridge Research Centre	18–22nd March 2019	CHO	18°06′44.5″ S 24°18′58.4″ E
		8–12th February 2020	CHO	
Okavango	Etsha	3–5th February 2020	OKA1	19°08′18.1″ S 22°20′04.1″ E
	Nxamasere	6–7th February 2020	OKA2	18°36′22.6″ S 22°03′59.1″ E
Central Kalahari Game Reserve	Matswere Gate	12th March 2019	CKGR1	21°09′23.4″ S 24°00′25.7″ E
	Sunday Pan	13th March 2019	CKGR2	21°19′58.3″ S 23°41′18.4″ E
	Kalahari Plains Lodge	14th March 2019	CKGR3	21°29′07.9″ S 24°00′30.1″ E
	Letiahau	15th March 2019	CKGR4	21°36′30.3″ S 23°37′05.4″ E
	Motopi	16th March 2019	CKGR5	21°07′57.9″ S 23°05′34.8″ E

**Table 3:** Mosquito identification information of species collected from major tourism destinations (Central Kalahari Game Reserve (CKGR), Okavango (OKA) and Chobe enclave (CHO)) in Botswana. Specimens were identified morphologically (morph.), molecularly (mol.) or both (morph/mol.) as described in the Materials and Methods section. The GenBank reference is given with pairwise identities (%). The GenBank corresponding reference, if available, is given whenever morphological identity is undefined at the species level.

Genus	Species	Nb. Specimens			Identification (morph/mol.)	GenBank ID (% Identity)
		CKGR	OKA	CHO		
*Aedes*	*Ae. aegypti*	1	0	2	morph/mol.	MK533633 (99.2–99.8)
	*Ae. mcintoshi*	0	3	0	mol.	LC473695 (97.9–99.8)
	*Ae.* sp. 1 ^1^	3	0	1	morph.	*-*
	*Ae.* sp. 2 ^1^	4	0	0	morph.	-
	*Ae.* sp. 3 ^1^	0	0	1	morph.	*-*
*Anopheles*	*An. coustani*	0	10	0	mol.	MK585951 (98.3–99.7)
	*An. gambiae* s.s	0	74	20	morph/mol.	NC_002084 (99.7–100)
	*An. squamosus* gp. 1	0	0	1	mol.	MK776750 (99.7)
	*An. squamosus* gp. 2	0	34	0	mol.	MK533644 (99.1)
	*An.* sp. 1	6	0	0	morph.	*An.* sp. 1 MT741511 (100)
	*An.* sp. 2 ^1^	0	1	0	morph.	-
	*An.* sp. 3	0	1	0	morph.	*An.* sp. Mali 1 MK585979 (99.4)
	*An.* sp. 4	0	1	0	morph.	*An.* sp. 15 MK776739 (98.7)
*Coquillettidia*	*Cq. fuscopennata*	0	111	0	morph/mol.	LC473712 (98.3–99.1)
	*Cq. metallica*	0	4	0	morph/mol.	LC473709 (99.8)
	*Cq. microannulata*	0	2	0	morph/mol.	LC473713 (99.2–99.4)
*Culex*	*Cx. neavei/simpsoni*	0	229	1	morph/mol.	*Cx. neavei* LC473635 (97.4–98.6) *Cx. simpsoni* KU187061 (96.9–98.0)
	*Cx. mirificus*	0	1	0	mol.	LC473643 (100)
	*Cx. perexiguus*	3	0	3	mol.	KU380423 (99.7–100)
	*Cx. pipiens*	26	10	131	morph/mol.	MZ206334 (99.7–100)
	*Cx. poicilipes*	0	164	0	morph/mol.	LC473618 (98.2–99.7)
	*Cx. rima*	0	111	0	morph/mol.	KU380462 (99.2–99.7)
	*Cx. simpliciforceps* ^2^	0	0	3	morph.	*Cx.* sp. LC507872 (98.8–99.1)
	*Cx. wigglesworthi* ^2^	0	0	1	morph.	*Cx. rima* LC473615 (97.3)
	*Cx.* sp. 1 ^1^	3	0	8	mol.	*Cx.* sp. 16GH LC507872 (98.9–99.8) *Cx. cinereus* LC473617 (97.0)
	*Cx. univittatus* ^3^	1	8	1	morph/mol.	LC102144 (99.0–99.7)
*Mansonia*	*Ma. africana*	0	1290	0	morph/mol.	KU380478 (98.5–99.1)
	*Ma. uniformis*	0	3207	0	morph/mol.	KU187168 (99.8–100)
*Mimomyia*	*Mi. mimomyiaformis*	0	1	0	mol.	LC473719 (100)
	*Mi. splendens*	0	2	0	morph/mol.	KU380391 (99.0–99.1)
*Uranotaenia*	*Ur.* sp. 1 ^1^	0	1	0	morph.	*-*
	*Ur.* sp. 2 ^1^	0	1	0	morph.	*-*
TOTAL		47	5266	173		

^1^ Specimens unidentified morphologically with no or weak match in GenBank. ^2^ Morphologically identified but species absent from GenBank. ^3^ Specimens from CKGR and CHO with bad sequences, morphological identification.

**Table 4:** Summary of mosquito species identified from touristic areas (Chobe enclave, Okavango and the Central Kalahari Game Reserve) and their main associated diseases in preferred hosts including humans (H), livestock (L) and wildlife (W).

Species	Main Associated Diseases	Preferred Hosts: Humans (H), Livestock (L), Wildlife (W)	References
*Aedes aegypti*	Dengue, yellow fever, Zika, Chikungunya Lumpy skin disease	H, L, W	[46,47]
*Aedes mcintoshi*	Rift Valley fever	L, W	[48]
*Anopheles coustani*	Human malaria Rift Valley fever, West Nile fever, Chikungunya	H, L, W	[49]
*Anopheles gambiae* s.s	Human malaria	H, L, W	[50,51,52]
	Rift Valley fever Cat-flea typhus Spotted fever		
*Anopheles squamosus* gp. 1 *Anopheles squamosus* gp. 2	Human malaria Rift Valley fever	H, W	[53]
*Coquillettidia fuscopennata*	Avian malaria, Chikungunya, yellow fever, Sindbis fever	H, W	[54,55,56]
*Coquillettidia metallica*	Avian malaria, West Nile fever, Rift Valley fever	H, W	[54,55,56]
*Coquillettidia microannulata*	Avian malaria	W	[55,56]
*Culex naevei*	West Nile fever, avian malaria	W	[55,57]
*Culex perexiguus*	West Nile fever, avian malaria	H, W	[58]
*Culex pipiens*	Rift Valley fever, West Nile fever, Filariasis, Encephalitis, Avian malaria	H, L, W	[50,58]
*Culex mirificus*	Lumpy skin disease	L, W	[47,59]
*Culex poicilipes*	Rift Valley fever, avian malaria	L, W	[55,57]
*Culex rima*	West Nile fever, encephalitis, avian malaria, filariasis	H, L, W	[60]
*Culex simpliciforceps*			[50,55]
*Culex* sp. 1	Rift Valley fever, avian malaria	H, L, W	
*Culex* sp. 2			
*Culex univittatus*	West Nile fever, Wesselsbron	H, L, W	[54]
*Mansonia africana*	Rift Valley fever, lymphatic filariasis, West Nile fever	H, W	[50,54,61,62]
*Mansonia uniformis*	Rift Valley fever, lymphatic filariasis, Avian malaria, West Nile fever, Zika, Chikungunya	H, W	[50,54,55,61,62]
*Mimomyia mimomyiaformis* *Mimomyia splendens*	West Nile fever	H, W	[63]
*Uranotaenia* sp. 1 *Uranotaenia* sp. 2	Wesselsbron	L	[54]

## Data Availability

The datasets during and/or analysed during the current study are available from the corresponding author on reasonable request.
